# Halo artifacts of indwelling urinary catheter by inaccurate scatter correction in ^18^F-FDG PET/CT imaging: incidence, mechanism, and solutions

**DOI:** 10.1186/s40658-020-00333-8

**Published:** 2020-11-13

**Authors:** Keiichi Magota, Naoto Numata, Daiki Shinyama, Junya Katahata, Yamato Munakata, Piotr J. Maniawski, Kentaro Kobayashi, Osamu Manabe, Kenji Hirata, Ukihide Tateishi, Kohsuke Kudo, Tohru Shiga

**Affiliations:** 1 Division of Medical Imaging and Technology, Hokkaido University Hospital, Sapporo, Japan; 2 Philips Japan, Tokyo, Japan; 3 Department of Diagnostic Imaging, Hokkaido University Graduate School of Medicine, Kita 15 Nishi 7, Kita-ku, Sapporo, Hokkaido 060-8638 Japan; 4 Philips Healthcare, Cleveland, Ohio USA; 5 Department of Diagnostic Radiology and Nuclear Medicine, Graduate School, Tokyo Medical and Dental University, Tokyo, Japan; 6 Global Station for Quantum Medical Science and Engineering, Global Institution for Collaborative Research and Education, Hokkaido University, Sapporo, Japan

**Keywords:** Halo artifact, Scatter correction, Urinary catheter, PET/CT, ^18^F-FDG

## Abstract

**Background:**

Halo artifacts from urinary catheters can occur due to inaccurate scatter correction, and the artifacts affect the tumor visibility in ^18^F-FDG PET/CT images. We investigated the incidence rate and the mechanisms of halo-artifact generation and explored several scatter correction techniques to prevent artifacts.

**Methods:**

We conducted patient and phantom studies. (1) We retrospectively reviewed the cases of patients who had undergone ^18^F-FDG PET/CT scans. To determine the frequency of halo-artifact generation, we used the patients’ PET images with a standard scatter correction based on a tail-fitted single-scatter simulation (TF-SSS) using 4-mm voxel μ-maps (TFS 4-mm). (2) We performed phantom studies to evaluate the effects of a urine catheter and two scatter correction techniques, i.e., TF-SSS with 2-mm voxel μ-maps (TFS 2-mm) and a Monte Carlo-based single-scatter simulation (MC-SSS) using 4-mm voxel μ-maps (MCS 4-mm). The average standardized uptake values (SUVs) were measured for axial PET images. (3) Using the patients’ data, we investigated whether TFS 2-mm and MCS 4-mm can eliminate the artifacts in the clinical images.

**Results:**

(1) There were 61 patients with urinary catheters; in five (8.2%), halo artifacts were observed in the TFS 4-mm PET images. (2) The phantom study clearly reproduced the halo artifacts in the TFS 4-mm PET images. The halo artifacts were generated when urine moved in the interval between the CT and PET imaging, and when the urinary catheter was placed in a circular shape. The SUVs for the TFS 4-mm and TFS-2mm PET images were underestimated at the halo-artifact regions, whereas the SUVs for the MCS 4-mm PET images were close to the true values. (3) The halo artifacts disappeared in the TFS 2-mm PET images in 4/5 patients but not 1/5 patient, whereas the halo artifacts were completely absent in the MCS 4-mm PET images in 5/5 patients.

**Conclusions:**

These data suggest that halo artifacts are caused if the PET images do not correspond to the physical material in the μ-maps, which induces the scatter correction error. With the MC-SSS, it was possible to accurately estimate the scatter without generating halo artifacts.

**Supplementary Information:**

**Supplementary information** accompanies this paper at 10.1186/s40658-020-00333-8.

## Introduction

Positron emission tomography/computed tomography (PET/CT) is useful for the assessment and management of many types of cancer including their diagnosis, staging, prognosis, and therapeutic efficacy. ^18^F-fluorodeoxyglucose (FDG) PET/CT in particular has become an important tool for identifying new and effective therapies in cancer treatment and for its role as an imaging biomarker [1, 2].

The main excretion pathway of ^18^F-FDG is through the kidneys, ureters, and bladder, and this makes it difficult to adequately visualize pelvic tumors by using filtered back-projection images because streak artifacts may appear in the pelvis. Such streak artifacts can be reduced by an iterative reconstruction algorithm [3], but when other radiotracers with renal excretion are used (e.g., ^68^Ga-labeled prostate-specific membrane antigen [PSMA]) [4], photopenic artifacts surround the kidneys and the bladder even in iterative reconstruction images [5, 6]. These are so-called halo artifacts that could potentially mask primary tumors and local recurrences.

The appearance of a halo artifact is caused by inaccurate scatter correction in the most commonly used algorithm [7, 8], which is a tail-fitted single-scatter simulation (TF-SSS) [9–11]. In ^68^Ga-PSMA PET/CT imaging, scatter correction becomes inaccurate due to errors in both the scatter estimate by the single-scatter simulation (SSS) and the scaling factor calculation by a fitting process [7, 8]. This type of halo artifact is reduced or eliminated by using the modified TF-SSS algorithm [7, 8, 12]. The possibility of the halo artifact’s appearance can also be reduced by oral hydration of the imaged patient, and by suppressing the patient’s urination urge by the administration of a diuretic before PET imaging [13], and by urine drainage using an indwelling urinary catheter into the bladder. This procedure is particularly important for the identification of pelvic tumors such as colorectal and bladder cancers [14, 15].

We have observed an artifact that is different from those caused by scatter correction error in the ^18^F-FDG PET/CT images for patients with an indwelling urinary catheter. This artifact is a type of halo artifact that appears in the region where the urinary catheter is present. A urinary catheter is positioned outside of the patient’s body, and we thus hypothesized that PET images may be affected by some scatter correction error because the region outside the patient’s body is masked in the TF-SSS algorithm. To the best of our knowledge, this artifact has not been comprehensively studied or reported. Because the artifact obliterates the true ^18^F-FDG uptake, the presence of a malignant tumor may not be detected, and an accurate quantitative evaluation such as measurement of the standardized uptake value (SUV) can be impossible.

We conducted the present study to determine the presence and rate of halo artifacts and to provide clinical examples. We retrospectively evaluated the cases of patients with indwelling urinary catheters who had undergone ^18^F-FDG PET/CT scans, and to clarify the mechanism underlying the artifacts, we performed phantom studies simulating ^18^F-FDG PET/CT imaging with urinary catheters. We propose steps that can be taken to eliminate halo artifacts by considering the μ-map voxel size in the TF-SSS algorithm and by applying a Monte Carlo-based single-scatter simulation (MC-SSS), which is the vendor’s own algorithm. We also investigated whether this approach can be applied to clinical patient data. The study was conducted to better understand halo artifacts and to explore the use of different scatter correction algorithms in order to reduce the generation of halo artifacts in the ^18^F-FDG PET/CT imaging of patients with an indwelling urinary catheter.

## Materials and methods

A Gemini TF PET/CT scanner (Philips Healthcare, Cleveland, OH, USA) was used for all imaging [16]. The coincidence and energy windows were used at their fixed settings of 3.8 ns and 460–665 keV, respectively. The acquisition was in three-dimensional mode only.

We conducted the patient and phantom studies to investigate the presence, rate, and underlying mechanisms of halo-artifact generation by using different scatter correction techniques as follows. (1) The frequency of halo-artifact generation was investigated using the ^18^F-FDG PET images with a standard scatter correction based on the TF-SSS using 4-mm voxel μ-maps (TFS 4-mm). (2) We performed phantom studies to evaluate the effects of a urine catheter and different scatter correction techniques, i.e., TF-SSS with 2-mm voxel μ-maps (TFS 2-mm) and the MC-SSS using 4-mm voxel μ-maps (MCS 4-mm). (3) We conducted an analysis of the patients’ data to determine whether the use of TFS 2-mm and/or MCS 4-mm can eliminate the artifacts in the clinical images.

### Patient study 1

We retrospectively analyzed patient data to identify the characteristics of halo-artifact generation and to quantify the rate of artifacts. The evaluations included all of the whole-body ^18^F-FDG PET/CT data of the patients with indwelling urinary catheters who were scanned at our institution during the period from June 2013 to January 2020.

The PET/CT scans were performed following our standard protocol: ^18^F-FDG (4.5 MBq/kg) was injected after a ≥ 6-h fast by the patient, although oral hydration with glucose-free water was allowed. For each patient, CT was performed for attenuation and scatter corrections, and then a PET scan was performed from the mid-femur or toes to the top of the head. The PET acquisition time was 1–2 min/bed position.

For the reconstruction of the PET images, the three-dimensional blob-based iterative list-mode ordered-subset expectation maximization algorithm with time-of-flight information was used under the following conditions: iterations, 3; subsets, 33; blob increment, 2.0375 voxels; blob radius, 2.5 voxels; blob shape parameter alpha, 8.3689; relaxation parameter, 0.7. All PET images were corrected for attenuation and scatter by using μ-maps converted from the CT images and resampled to isotropic 4-mm voxels, which matched the μ-maps. The scatter correction algorithm was TF-SSS (TFS 4-mm) [11, 17], which is the default setting in this scanner. Images with attenuation correction but non-scatter-correction (NSC) were also reconstructed. The image matrix size was 144 × 144 pixels for the 576-mm field of view (FOV). All reconstructions were performed offline using Philips PET reconstruction software. The parameters for the CT imaging were as follows: tube voltage, 120 kVp; tube current, 200 mAs; FOV, 600 mm.

In the visual assessment, we excluded halo artifacts that were due to movements of the patients’ arm(s) or diaphragm by comparing PET images without attenuation/scatter corrections and the CT images. Images with the urinary catheter located outside of the transverse PET FOV were also excluded.

We defined the halo artifacts caused by a urinary catheter in the following two steps: (1) we identified cold regions by detecting consecutive zero-valued voxels in the PET images, and then (2) we verified whether the cold regions were not present in the NSC PET images. When halo artifacts were present, we manually set the volume-of-interest to the entire trunk and measured the mean standardized uptake value (SUV). The evaluation was performed by a single reader using the IntelliSpace Portal (ver. 5.0.0.20030; Philips Healthcare). We also obtained prompt and random events sinograms, scatter sinograms by TFS 4-mm, and μ-map sinograms for a comparison of radial profiles. We defined a “mask” for the TFS 4-mm by thresholding the attenuation coefficients on the μ-map sinograms at a value lower than 1.005 cm^–1^.

### Phantom study

To explore the mechanism(s) underlying the halo artifacts and to devise improvements to eliminate the halo artifacts, we conducted an experiment to reproduce halo artifacts by using a body phantom from the National Electrical Manufacturers Association (NEMA) with the spherical inserts removed and with a urinary catheter made of 1-mm-thick vinyl chloride (7-mm inner dia., 9-mm outer dia.).

Referring to our findings in patient study 1, we identified the following two appearance patterns of halo artifacts.

**Fig. 1:**
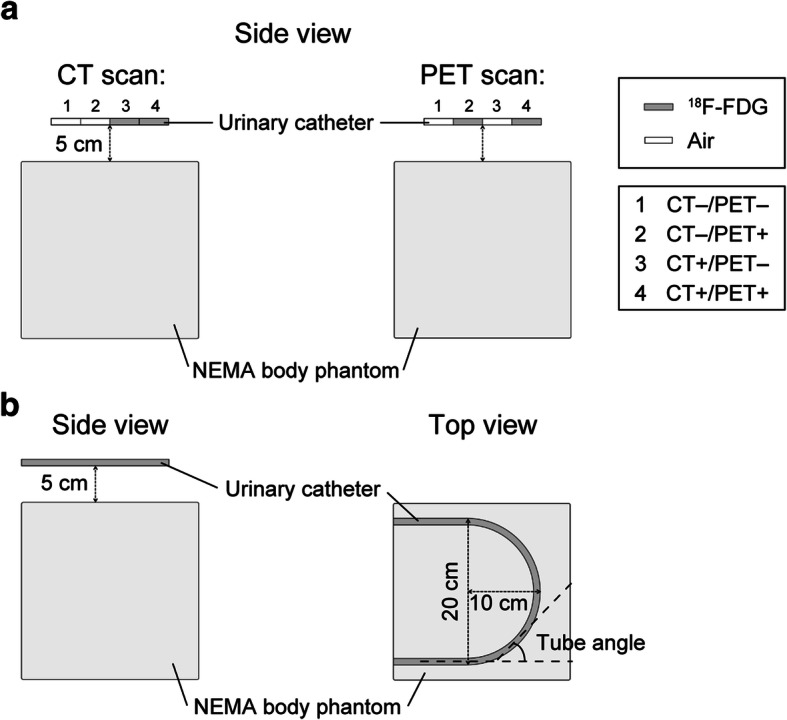
The phantom studies. Urine shift pattern (**a**). Tube curve pattern (**b**)

One pattern was observed in the cases in which there were urine shifts between the CT imaging and the subsequent PET imaging. This shift may have arisen as the urine in the urinary catheter flowed downward from the upper part of the urinary catheter after the CT imaging. This pattern (hereafter called the “urine shift pattern”) corresponds to the scenario in which urine is not present in the CT scan but appears in the PET scan (CT−/PET+) as in the phantom diagram of Fig. 1a.

For comparison, we also set regions at which urine appears in the CT scan but not in the PET scan (CT+/PET−), or no urine was present in either the CT or PET scan (CT−/PET−), or urine was present in both the CT and PET scans (CT+/PET+). The length of the urinary catheter for each region was 4 cm.

The second pattern was observed in the cases in which the urinary catheter was arranged in an arc (hereafter referred to as the “tube curve pattern”) and urine was present throughout the urinary catheter. Here, the urinary catheter was placed in a semicircle with a radius of 10 cm at a position 5 cm away from the NEMA body phantom (Fig. 1b). The urinary catheter and the NEMA body phantom were filled with 150 kBq/mL and 3.0 kBq/mL of ^18^F-FDG solution, respectively. The SUV inside the NEMA body phantom was adjusted to 1.0. The PET data were acquired at two bed positions (2 min/bed position).

The PET images were reconstructed under the same conditions as those in patient study 1, with the two additional scatter corrections, TFS 2-mm and MCS 4-mm. The “Discussion” section below and previous studies provide detailed descriptions of the MC-SSS algorithm [18, 19]. The CT imaging parameters were the same as those in patient study 1. Sinograms were also evaluated as in patient study 1; prompt and random events sinograms, the scatter sinograms provided by the use of TFS 2-mm and MCS 4-mm, and μ-map sinograms were obtained for a comparison of radial profiles. We manually set the volume-of-interest for the entire NEMA body phantom to measure the mean SUV for each slice.

### Patient study 2

Here, we applied two image reconstruction settings added in the phantom studies to patient cases in which halo artifacts appeared. We investigated the presence/absence of halo artifacts using the same evaluations as in patient study 1.

## Results

### Patient study 1

Of the 3633 whole-body ^18^F-FDG PET/CT cases at our institute, we identified 61 patients with urinary catheters. Table 1 summarizes the demographics of these patients. Of the 61 patients, five (8.2%) had halo artifacts caused by the urinary catheter in TFS 4-mm PET images.

**Table 1:** Background of the 61 patients with urinary catheters

Characteristics	Description
Age, years	61 (17–87)
Males/females	32/29
Body weight, kg	57 (28–90)
Blood sugar, mg/dL	117 (67–157)
^18^F-FDG injection dose, MBq	254 (128–415)
Diagnosis, no. of patients	
Malignant lymphoma	17
Brain tumor	10
Spinal tumor	10
Bladder cancer	6
Fever of unknown origin	6
Colorectal cancer	2
Lung cancer	2
Extramammary Paget disease	2
Cancer of unknown primary origin	2
Kidney cancer	1
Ovarian cancer	1
Laryngeal cancer	1
Subcutaneous tumor	1

Data are the number or average (range)

**Fig. 2:**
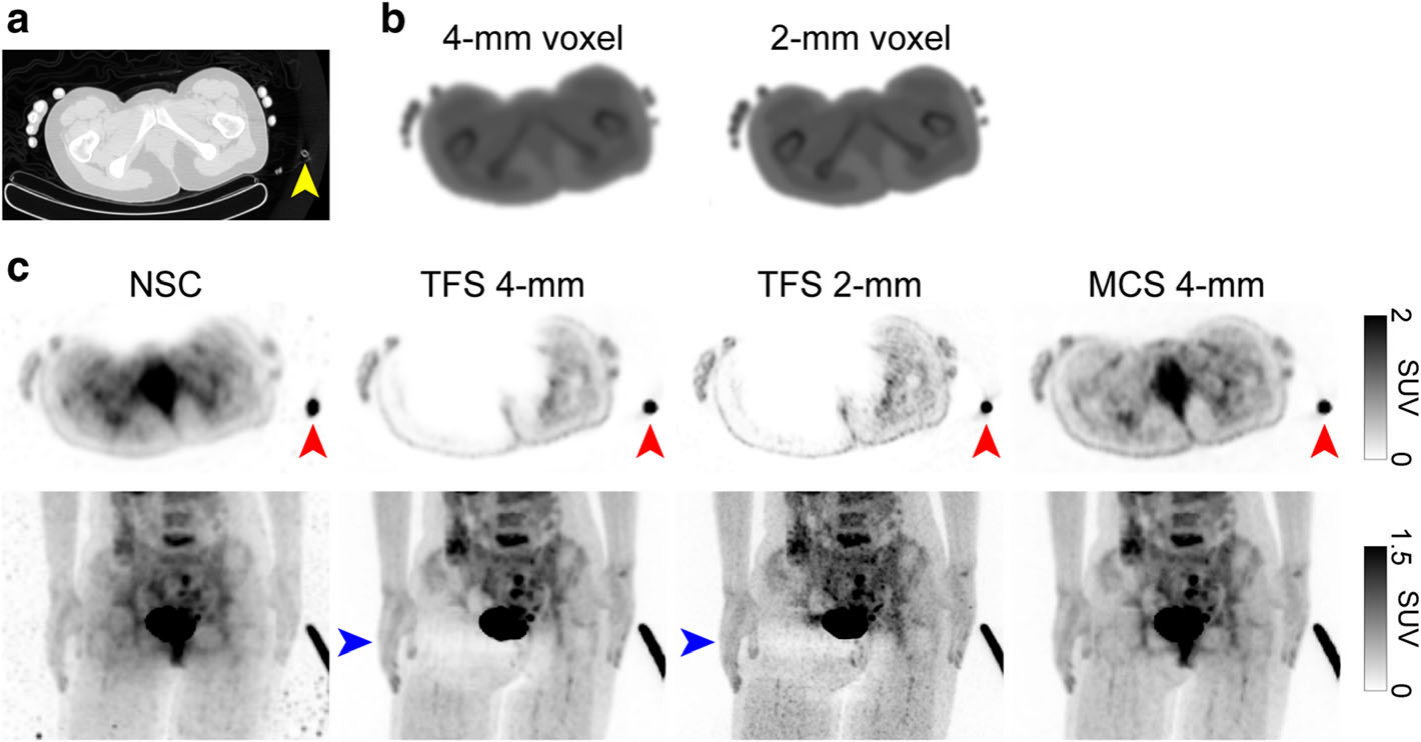
Representative PET images of a patient (urine shift pattern). CT image (**a**), 4-mm and 2-mm voxel μ-maps (**b**), PET images with non-scatter-correction (NSC), TFS 4-mm, TFS 2-mm, and MCS 4-mm (**c**). *Yellow arrow*: urinary catheter. *Red arrows*: urine in the urinary catheter. *Blue arrows:* halo artifacts

**Fig. 3:**
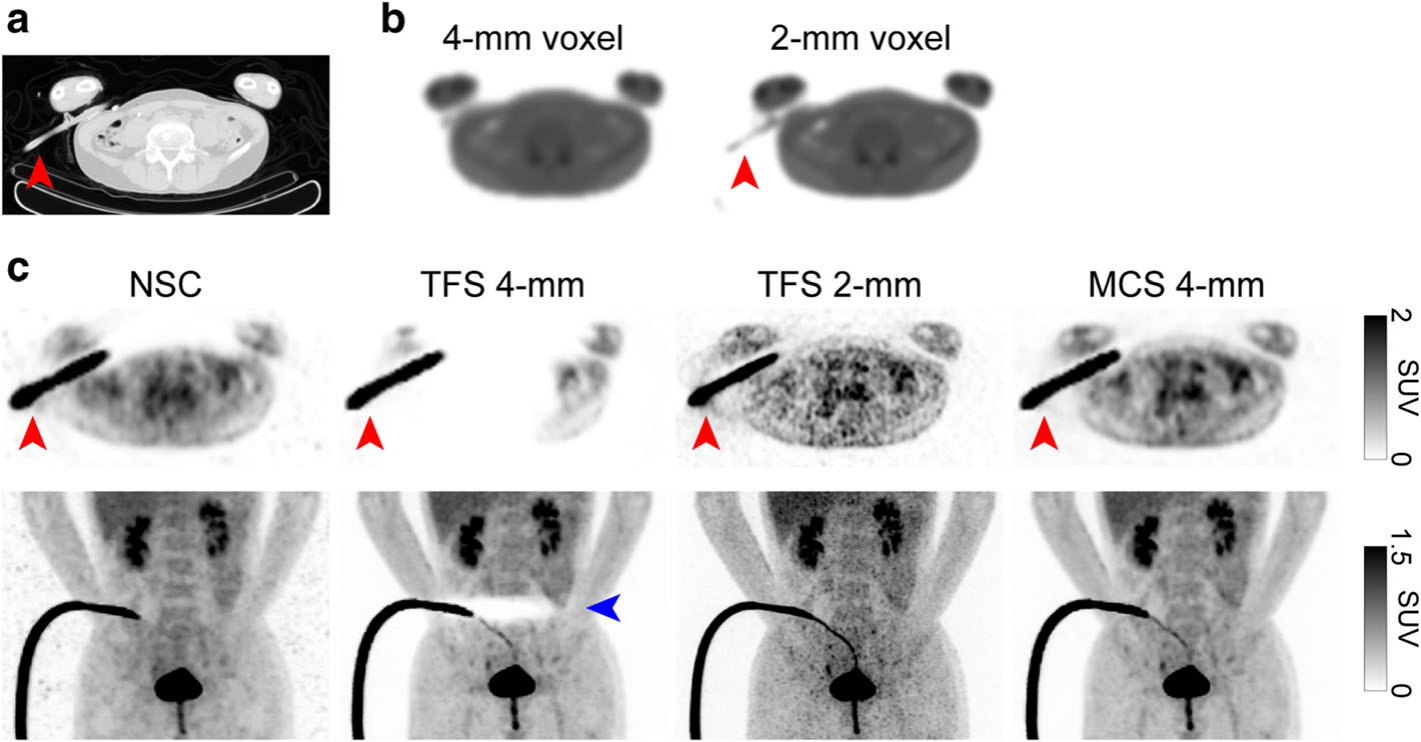
Representative PET images of a patient (tube curve pattern). CT image (**a**), 4-mm and 2-mm voxel μ-maps (**b**), PET images with non-scatter-correction (NSC), TFS 4-mm, TFS 2-mm, and MCS 4-mm (**c**). *Red arrows*: urinary catheter with urine. *Blue arrow*: halo artifact

**Fig. 4:**
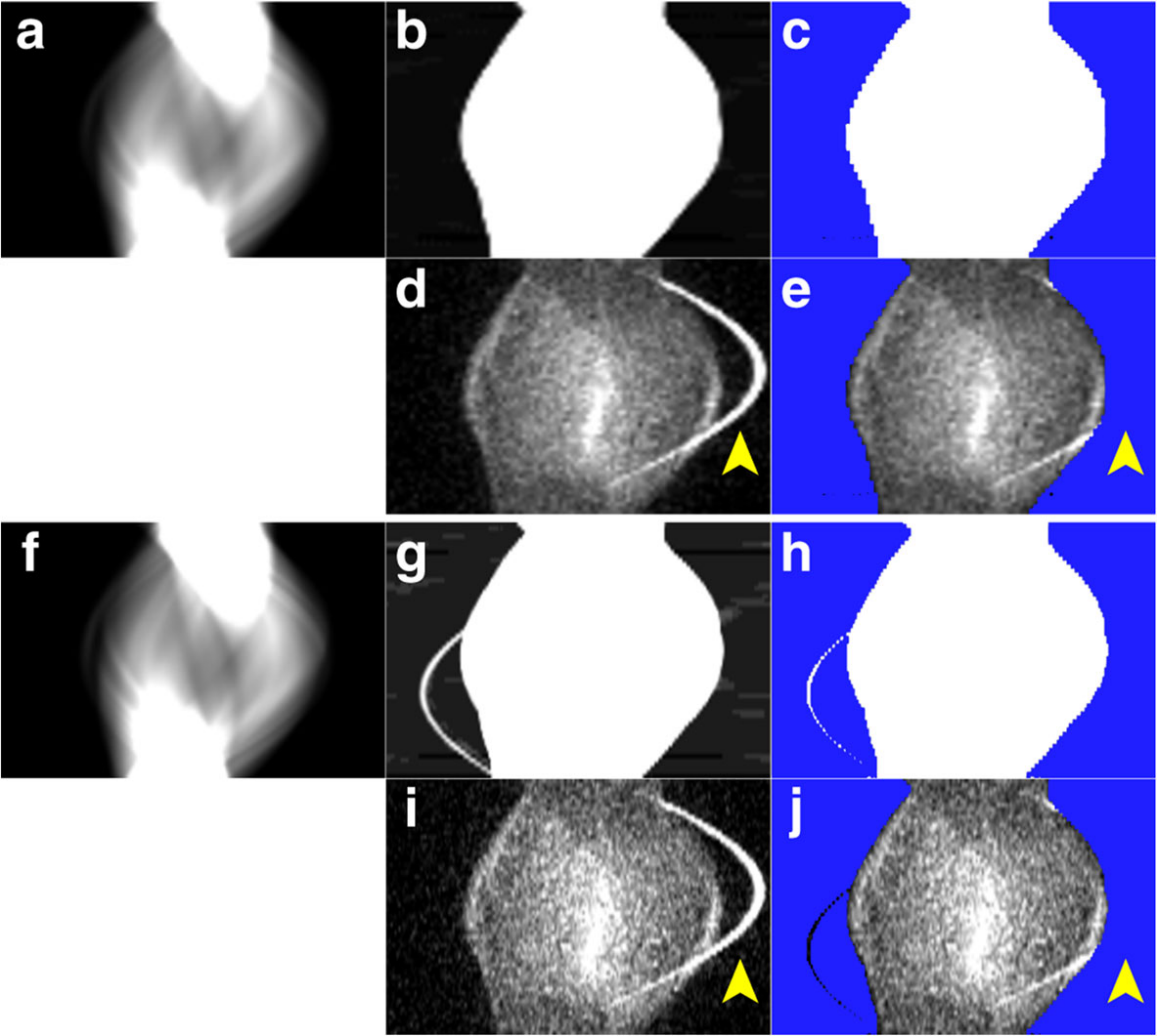
Sinogram images on 4-mm (**a**–**e**) and 2-mm (**f**–**j**) voxel size (urine shift pattern). μ-map sinogram (**a**, **f**), changing display scaling of **a**, **f** (**b**, **g**), mask for TF-SSS algorithm (**c**, **h** *in blue*), random-corrected emission (**d**, **i**), and mask superimposed on the emission sinogram (**e**, **j**). Both 4-mm and 2-mm voxel size μ-map sinograms masked the urinary catheters (*yellow arrows*)

**Fig. 5:**
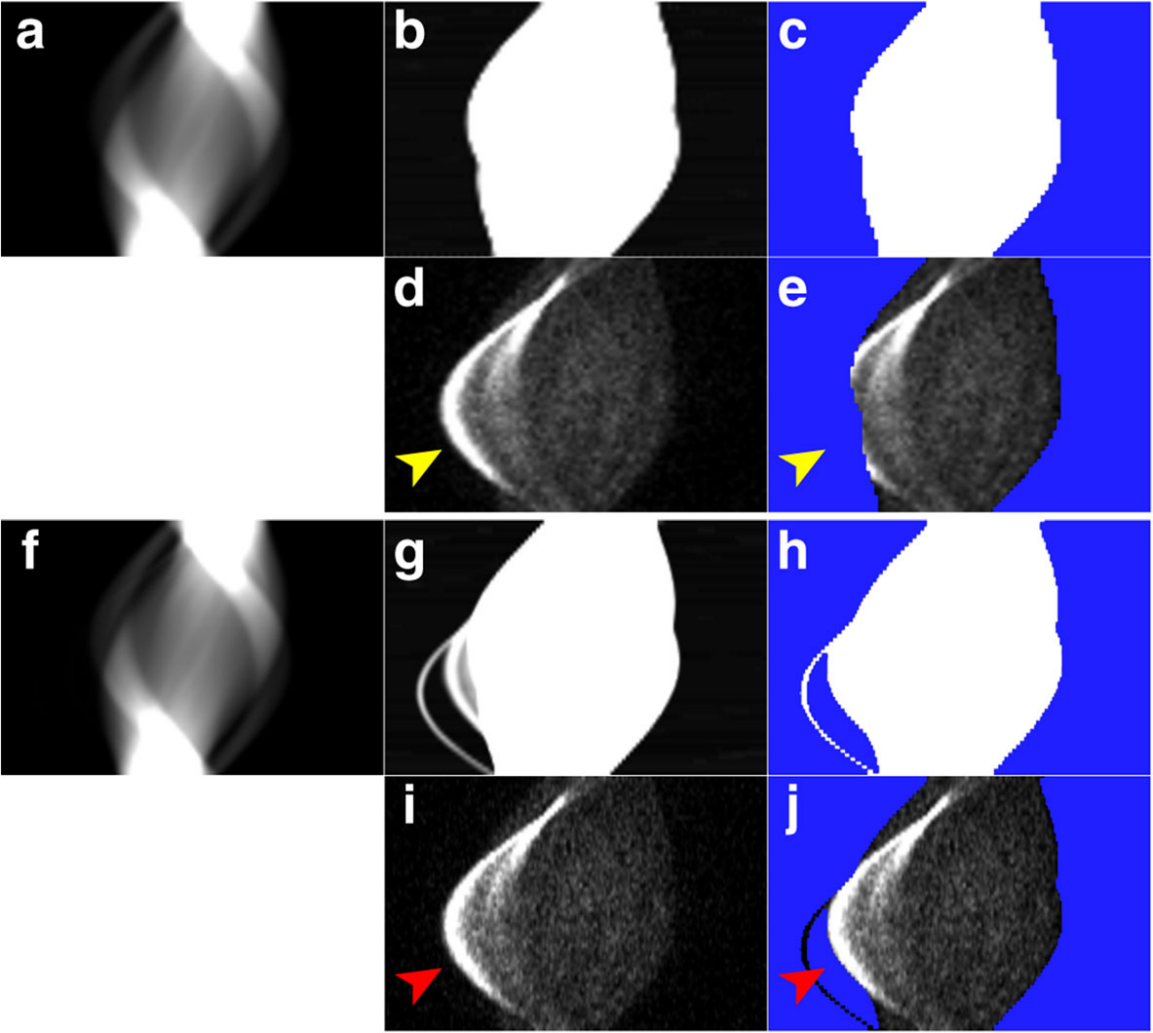
Sinogram images on 4-mm (**a**–**e**) and 2-mm (**f**–**j**) voxel size (tube curve pattern). μ-map sinogram (**a**, **f**), changing display scaling of **a**, **f** (**b**, **g**), mask for TF-SSS algorithm (**c**, **h** *in blue*), random-corrected emission (**d**, **i**), and mask superimposed on the emission sinogram (**e**, **j**). The urinary catheter was masked in the 4-mm voxel size μ-map sinogram (*yellow arrows*), but not in the 2-mm voxel size μ-map sinogram (*red arrows*)

**Fig. 6:**
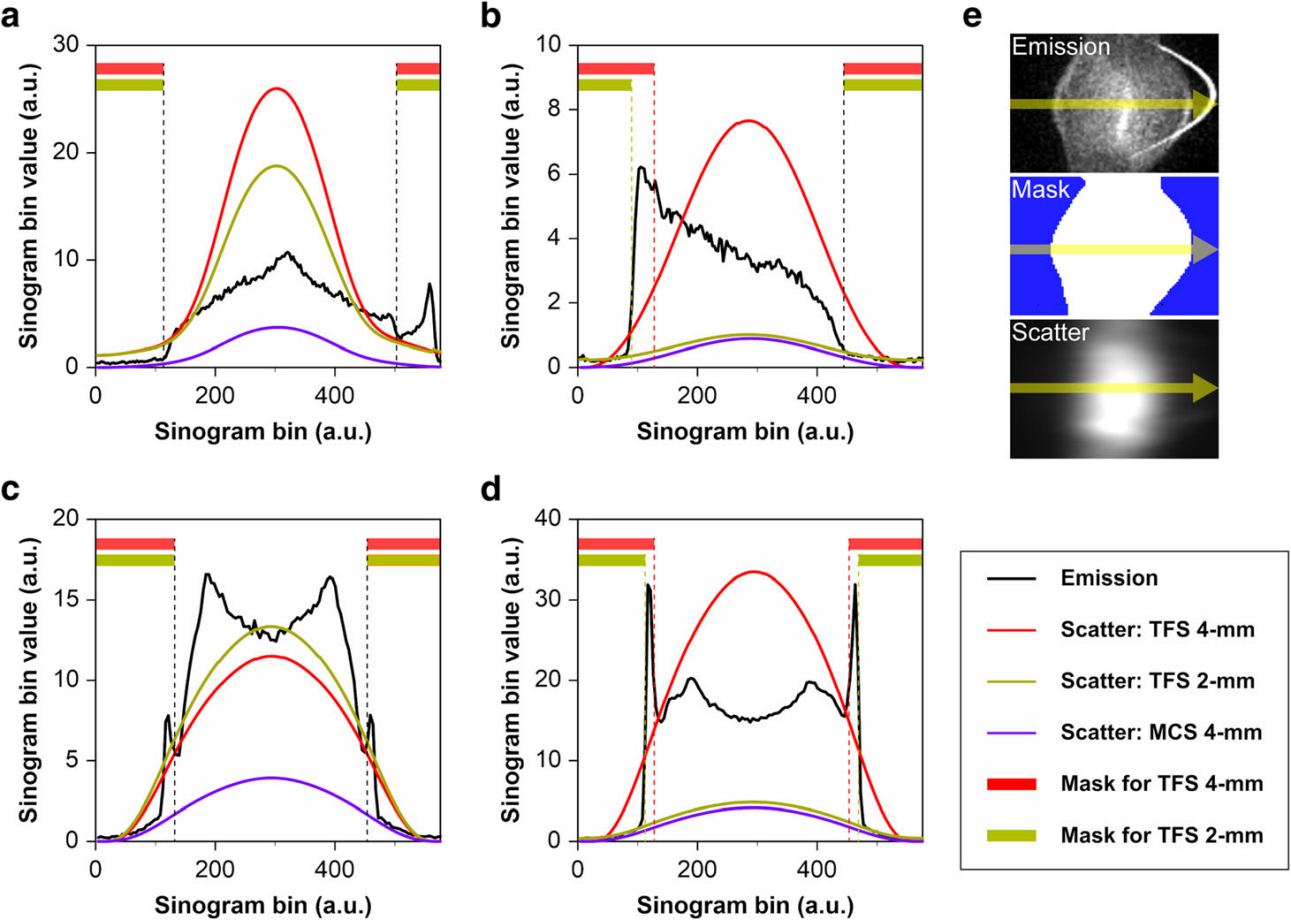
Sinogram profiles on halo-artifact regions from a random-corrected emission sinogram (*thick black solid line*), scatter sinogram (*red solid line:* TFS 4-mm, *green solid line*: TFS 2-mm, *blue solid line*: MCS 4-mm), and mask for TF-SSS (*red region*: for TFS 4-mm, *green region*: for TFS 2-mm). Patient data are shown in **a** and **b**. Phantom data are shown in **c** and **d**. Panels **a** and **c** are the urine shift pattern. Panels **b** and **d** are the tube curve pattern. **e** Example of sinogram images and profile lines

Figures 2 and 3 illustrate two representative cases. The sinogram images of μ-maps, a mask for TFS 4-mm, and emission (prompt-minus-random) are shown in Figs. 4a–e and 5a–e. In both cases, the urinary catheters were absent on the μ-map sinograms due to the masking process for TFS 4-mm (yellow arrows in Figs. 4b, c and 5b, c). Figure 6 shows the sinogram profiles corresponding to the horizontal-axis direction in Figs. 4 and 5. The scatter was overestimated (red lines in Fig. 6a, b), and thus a halo artifact was identified on the TFS 4-mm PET images, but no halo artifacts were present on the NSC images (Figs. 2c and 3c).

Our visual observation of the μ-maps of the two cases revealed that urinary catheters were positioned inside the masked regions and were absent on the 4-mm voxel μ-maps (Figs. 4b, c and 5b, c). The urine in the catheter was not visible in the CT image (yellow arrow in Fig. 2a), and not present on the μ-maps (Figs. 2b and 4b). However, the PET images showed the urine (red arrows in Fig. 2c). This suggests that there is a mismatch between the μ-maps and the PET images, and this mismatch may be ascribed to the urine in the urinary catheter having shifted in the interval between the CT imaging and the PET imaging (urine shift pattern).

Figure 3 shows the presence of a urinary catheter and urine in both the CT and PET images (red arrows in Fig. 3a, c), but the urinary catheter and urine are absent in the 4-mm voxel μ-map (Figs. 3b and 5b), leading to the mismatch between the μ-maps and the PET images. This mismatch occurred for a patient’s case with the urinary catheter placed in an arc (blue arrow in Fig. 3c). Four cases including the one illustrated in Fig. 3 showed a similar phenomenon, i.e., the tube curve pattern.

### Phantom study

We developed an experimental approach to reproduce the phenomenon that occurred in patient study 1.

**Fig. 7:**
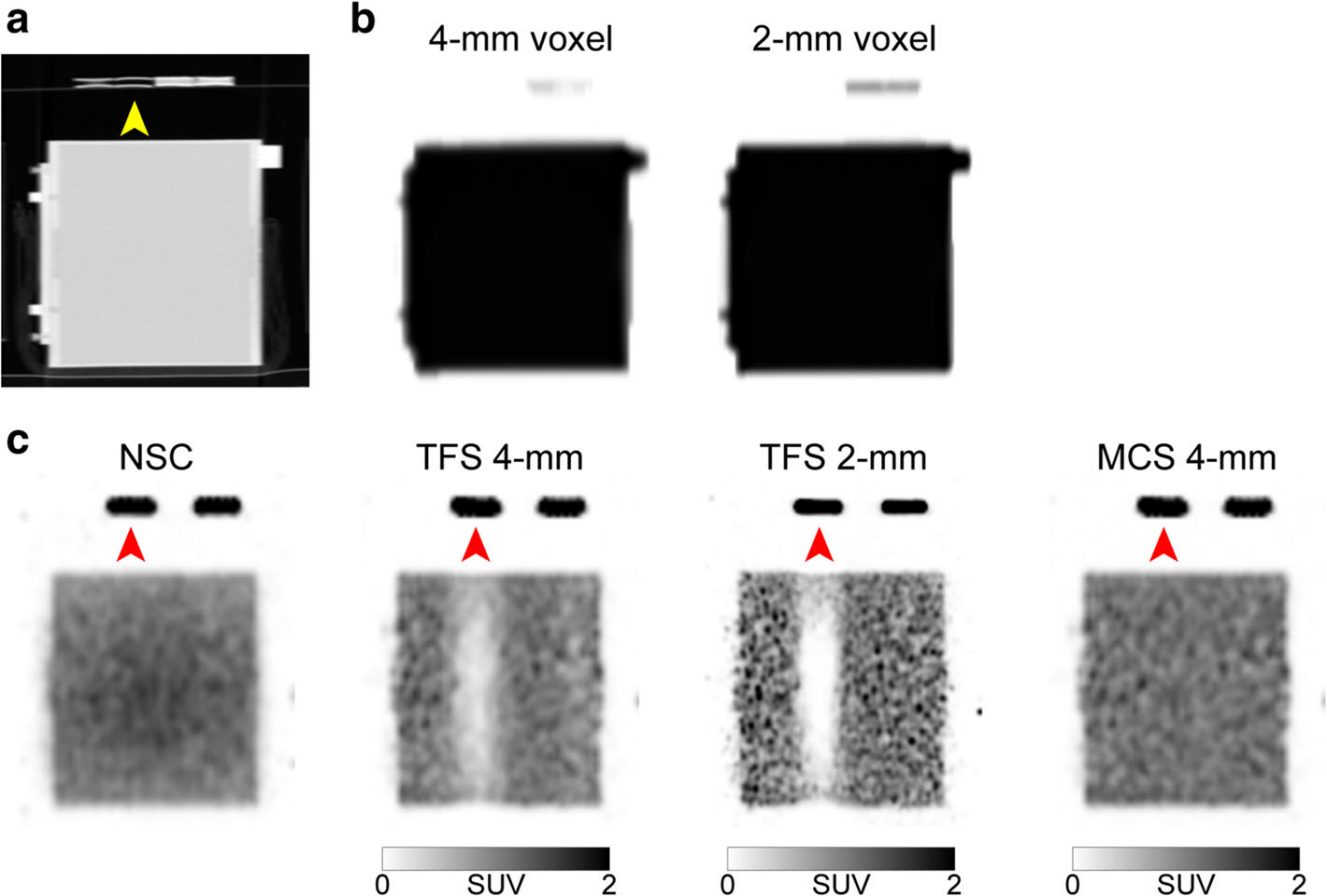
Sagittal images of a phantom (urine shift pattern). CT image (**a**), 4-mm and 2-mm voxel μ-maps (**b**), PET images with non-scatter-correction (NSC), TFS 4-mm, TFS 2-mm, and MCS 4-mm (**c**). *Yellow arrow*: urinary catheter in a CT−/PET+ region. *Red arrows*: ^18^F-FDG solution in a urinary catheter in a CT−/PET+ region

Figure 7 provides phantom images that simulate the urine shift pattern. The region with the mismatch between the CT images (μ-maps) and the PET images identified in patient study 1 corresponds to the second urinary catheter region from the left in Fig. 1a. No ^18^F-FDG solution was added to the urinary catheter that was showing in the CT image, and this urinary catheter was not visualized in the μ-maps. However, the PET images showed the ^18^F-FDG solution, suggesting that there is a mismatch between the μ-maps and the PET images, as in patient study 1.

**Fig. 8:**
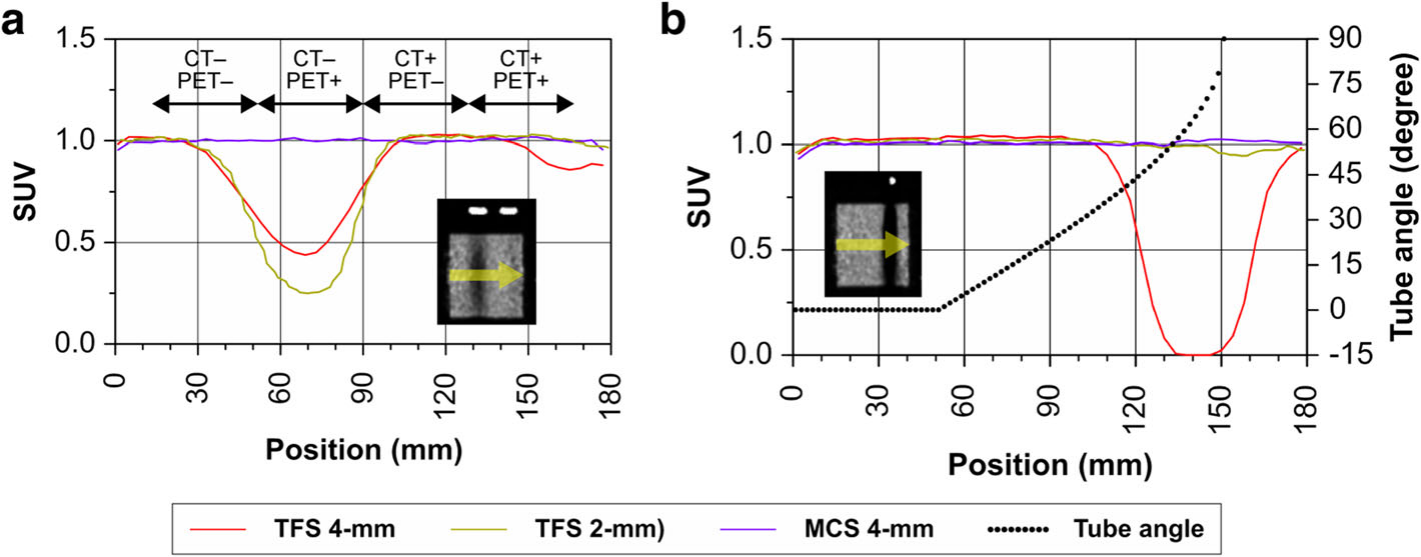
Average SUV per slice of phantom images for the urine shift pattern (**a**) and the tube curve pattern (**b**). The origin (*x*-axis) of the slice position corresponds to the left limit of the phantom in sagittal images from Figs. 7c and 9c (see images inside Graph). *Red line*: TFS 4-mm PET, *green line*: TFS 2-mm PET, *blue line*: MCS 4-mm PET. *Black dots* in **b** show the tube angle defined in Fig. 1b

The TFS 4-mm in the urine shift pattern overestimated the scatter (red line in Fig. 6c), resulting in the presence of halo artifacts. The SUV was < 0.5 and was underestimated (slices 30–100 in Fig. 8a). Halo artifacts also appeared in the TFS 2-mm PET image. The urinary catheter was not shown in the 2-mm voxel μ-map, causing the mismatch and the scatter to be overestimated (green line in Fig. 6c). By contrast, in the MCS 4-mm PET image, the scatter was correctly estimated (blue line in Fig. 6c) and no artifact appeared, resulting in a correct evaluation of the SUV (Fig. 8a). No artifact appeared in other regions (CT−/PET−, CT+/PET−, CT+/PET+) with the scatter accurately estimated, and the SUV at these regions was 1.0 (Fig. 8a, Suppl. Fig. S1).

**Fig. 9:**
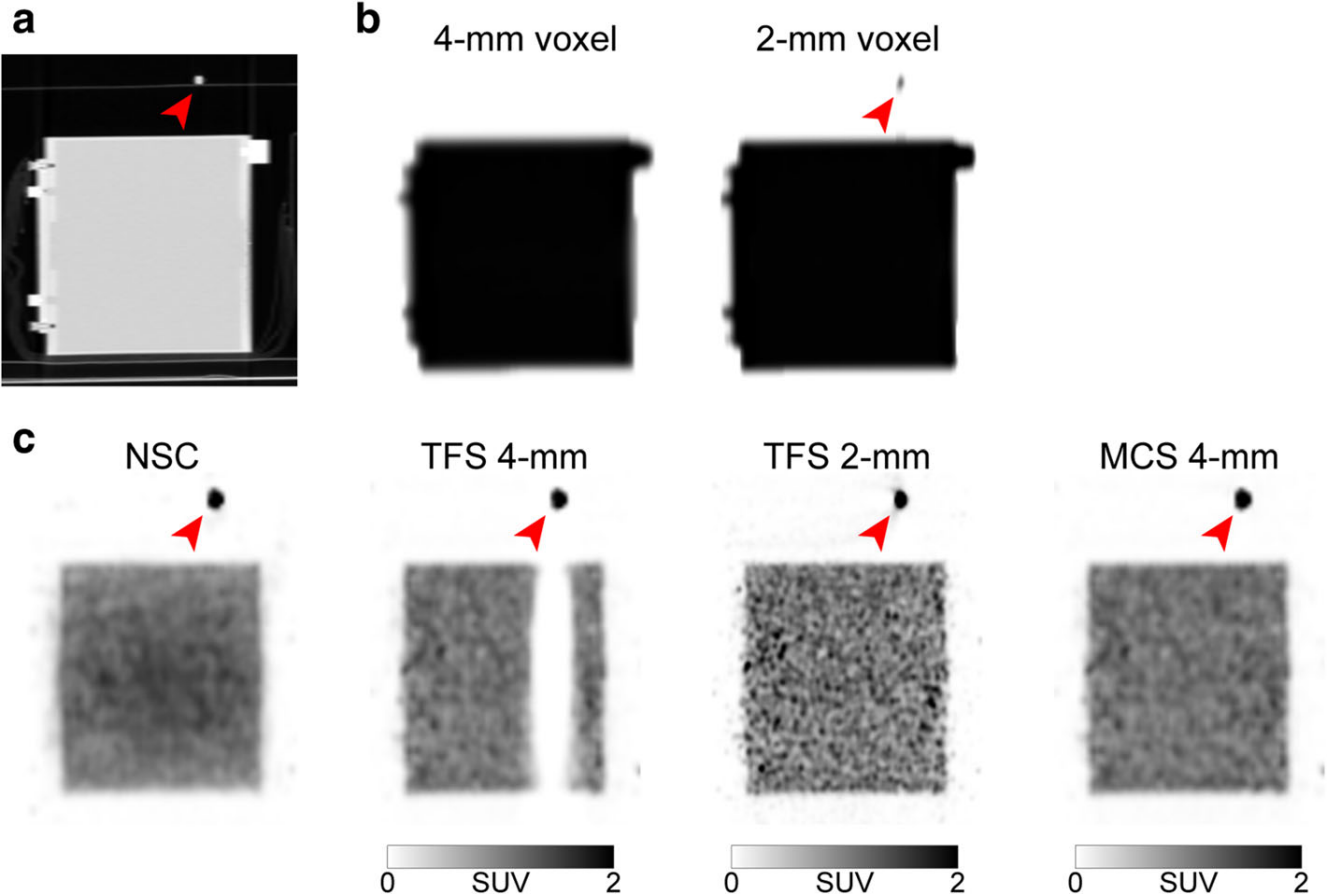
Sagittal images of a phantom (tube curve pattern). CT image (**a**), 4-mm and 2-mm voxel μ-maps (**b**), PET images with non-scatter-correction (NSC), TFS 4-mm, TFS 2-mm, and MCS 4-mm (**c**). *Red arrows*: ^18^F-FDG solution in the urinary catheter

Figure 9 provides phantom images that simulate the tube curve pattern. The region of the arc-shaped urinary catheter appears in the CT image but not on the 4-mm voxel μ-map. This resulted in mismatch with the PET image. The TFS 4-mm overestimated the scatter (red line in Fig. 6d), resulting in the presence of halo artifacts. The SUV was close to zero and was underestimated when the tube angle was large (slices 110–180 in Fig. 8b). By contrast, no halo artifact appeared in the TFS 2-mm PET image. Here, the urinary catheter was clearly depicted in the 2-mm voxel μ-map, and the scatter was correctly evaluated (green line in Fig. 6d). In the MCS 4-mm PET image, the scatter was also correctly evaluated (blue line in Fig. 6d), and no halo artifact appeared. Since there was no halo artifact, the SUVs for both TFS 2-mm and MCS 4-mm PET images were 1.00 ± 0.05 (mean ± standard deviation) in all catheter regions (Fig. 8b).

### Patient study 2

We investigated whether the artifacts could be improved by using TFS 2-mm or MCS 4-mm in cases in which halo artifacts had appeared in the TFS 4-mm PET images.

In the urine shift pattern, the urinary catheter was not depicted in the 2-mm voxel μ-map (Figs. 2b and 4g, h), and there was a mismatch between the μ-maps and the PET images as with the TFS 4-mm PET images. The result was that the scatter was overestimated, and the halo artifact remained (Figs. 2c and 6a).

In the tube curve pattern, the urinary catheter was depicted in the 2-mm voxel μ-map, and this eliminated the mismatch (Fig. 3b and 5g, h). The result was that the scatter was correctly evaluated, and no halo artifact appeared here (Figs. 3c and 6b).

The MCS 4-mm image also correctly evaluated the scatter in both patterns, and there were no halo artifacts (Figs. 2c, 3c, and 6a, b).

Table 2 summarizes the SUV data for the five patient cases with halo artifacts. In the cases with the urine shift pattern, the SUV of the TFS 4-mm PET image was similar to the TFS 2-mm PET image, but that of the MCS 4-mm PET image was larger than both TF-SSS PET images. In the cases with the tube curve pattern, the average SUVs of the TFS 2-mm and MCS-4mm PET images were larger than those of the TFS 4-mm PET images.

**Table 2:** Standard uptake values of 5 patients with halo artifacts

	TFS 4-mm PET	TFS 2-mm PET	MCS 4-mm PET
Urine shift pattern (*n* = 1)	1.15	1.17	1.56
Tube curve pattern (*n* = 4)	0.41 ± 0.06	0.67 ± 0.18	0.69 ± 0.17

The halo artifacts disappeared in the TFS 2-mm PET images in four of the five patients but not in the remaining patient, whereas the halo artifacts were completely absent in the MCS 4-mm PET images in all five patients.

## Discussion

We have described halo artifacts caused by an indwelling urinary catheter in ^18^F-FDG PET/CT imaging that can potentially complicate tumor assessments in artifact-hampered regions. The results of our patient and phantom studies demonstrated that scatter correction errors are a cause of halo artifacts. These results established that the halo artifacts appear in the TF-SSS PET images when there is a mismatch between the μ-maps and the PET images due to a shift in urine shift or a curve in the catheter tube. The halo artifacts were improved when the μ-map voxel size was reduced, but the halo artifacts remained in one case. With the MC-SSS method, it was possible to conduct accurate scatter correction without generating halo artifacts in all cases, indicating that it is possible to improve the accuracy of the diagnoses of tumor presence and quantitative analyses.

The scatter correction algorithm commonly used in PET/CT scanners is the TF-SSS method. An SSS is an analytical method to estimate the scatter contribution derived from the activity distribution and attenuation map. However, the scatter contribution from multiple scatters and activities located outside the FOV are not considered. The scaling factor that is estimated using the tail part on the scatter sinogram is assumed to include only the contribution of scatter. The scatter contribution determined by the SSS needs to be scaled to match the measured sinogram [11]. The tail part on the sinogram is identified by a mask from the μ-maps. Therefore, obtaining accurate scatter corrections by the TF-SSS depends on whether it is possible to accurately detect the tail part on the μ-maps. As shown in this study, the identification of the tail part is inaccurate when there is a mismatch between the μ-maps and PET images. Consequently, errors occur in the fitting process, and the scatter will be overestimated only if the radioactivity in the PET images does not correspond to the physical material in the μ-maps.

A mask for identification of the tail part on the μ-map sinograms is created by thresholding at a value lower than 1.005 cm^−1^, and urinary catheters are made of 1-mm-thick vinyl chloride with a low attenuation coefficient (1.03 cm^−1^). Many voxels less than thresholding value tend to be generated because the attenuation coefficients of the urinary catheters became lower due to the partial volume effect, averaging with the air around the urinary catheters by downsizing to match the PET voxel size and Gaussian filtering [20]. This scenario is particularly likely to occur when the urinary catheter is positioned three-dimensionally in an oblique direction with respect to the data matrix (like the tube curve pattern) and the large voxel size of the μ-maps (Figs. 3b and 9b).

In the 2-mm voxel μ-maps, the influence of the partial volume effect is reduced, and the urinary catheter would be depicted. However, as shown in our phantom study, the urinary catheter was depicted and did not generate the halo artifact when the catheter was positioned on an almost straight line with respect to the data matrix, even in the TFS 4-mm PET images (slices 0–90 in Fig. 8b). This was the situation for a large fraction of the patient cases which did not show any halo artifact.

It has been reported that artifacts in ^15^O-gas brain PET imaging are caused by a phenomenon similar to that observed herein [19]. This arises from the mismatch between the μ-maps and PET images when the face mask that is used to collect the patient’s exhaled breath containing ^15^O-gas has “disappeared” from the μ-maps [19].

When the scatter estimate exceeds the measured prompts, many pixels on the sinogram have negative values. In general, in an iterative reconstruction algorithm, negative values are converted to zero values due to sinogram non-negativity constraints. This is the cause of low SUVs in PET images with halo artifacts.

An MC-SSS is a combination of the SSS with the technique of scaling the results of the SSS using the scaling factor calculated by a Monte-Carlo simulation [18, 19]. In the calculation of this scaling factor, only the ratio of the total scattered events and the total true plus scattered events is required. Because there is no need to identify the tail part on the μ-maps, this technique results in an absence of artifacts even with a mismatch between the μ-maps and PET images.

In the TFS 4-mm PET images of the urine shift pattern, we observed that the SUV was slightly underestimated at the slice positions 150–180 (Fig. 8a). The reason for this underestimation could be because there was a mismatch between the μ-maps and PET images due to spillover from the CT+/PET+ region to locations without the urinary catheter (slices 165–180).

Although additional tests using PET/CT scanners from other vendors may be necessary, we speculate that the halo artifact could appear because the scatter correction algorithm used by many other vendors is the TF-SSS. The halo artifacts appear in the TF-SSS PET images when there is a mismatch between attenuation and activity images. Since the urine shift pattern is based on the physical mismatch between attenuation and activity images due to the downward flow of urine in the urinary catheter from the upper part of the catheter after the CT imaging, it is reasonable to speculate that the halo artifacts will appear on other PET/CT scanners. Our present findings revealed that the appearance of halo artifacts in the tube curve pattern depends on the voxel size of the μ-maps and the threshold value on the μ-map sinograms that is used for the identification of the tail part [17]. However, few details of the conversion process from the CT image to the μ-map have been published, and it is unclear whether the halo artifacts appear on PET/CT scanners from vendors other than Philips Healthcare (used herein).

A feasible method to prevent the appearance of halo artifacts in TF-SSS PET images is to make the PET scanner recognize that the body contour and the urinary catheter are located in the same spatial position by attaching the urinary catheter to the patient’s skin surface (Suppl. Fig. S2). However, even with this method, streak artifacts on the filtered back-projection images due to an accumulation of a sufficient amount of urine may interfere with the detection of a tumor [3]. An additional problem remains; the radiation exposure to the medical staff will increase due to the additional patient handling time.

The appearance of halo artifacts due to a urinary catheter is rare, but our present findings indicate that the influence of image defects due to halo artifacts is significant, as the artifacts can make it impossible to detect and quantify tumors. Fortunately, there were no patients in the present series for whom a tumor was masked by the halo artifact and for whom the quantification could not be accomplished. In clinical settings, it would be possible to diagnose the presence of a tumor at the region where halo artifacts have appeared by referring to NSC images. However, with NSC images, quantitative values such as the SUV cannot be measured. Scatter makes linearity between the radioactivity on the PET image and the true radioactivity concentration decrease. Scatter correction by an accurate estimation of scatter is therefore essential for quantitative PET images [21].

The MC-SSS algorithm does not generate the halo artifact even in the urine shift pattern. However, this algorithm is a vendor-specific method, and not all vendors implement this algorithm at present. If users cannot use the MC-SSS algorithm, we recommend the use of the TF-SSS algorithm with high resolution μ-maps (e.g., 2-mm voxels). That way, the halo artifacts would disappear in a significant number of patients. However, as shown in our study, the halo artifacts may remain in the urine shift pattern. Providing PET images without halo artifacts will reduce the possibility of false-negative diagnoses of tumors and will contribute to accurate quantitative analyses such as SUV measurements.

## Conclusion

Halo artifacts arose due to a mismatch between the μ-maps and PET images, and this mismatch induces errors in the scatter correction. With the TF-SSS method, the halo artifact was improved when the μ-map voxel size was reduced, but the artifacts remained in one case. With the MC-SSS method, it was possible to conduct an accurate scatter correction without generating halo artifacts in all cases, suggesting that it is possible to improve the accuracy of both diagnoses of tumor presence and quantitative analyses.

## Supplementary Information

**Additional file 1: Supplemental Fig. S1.** Sinogram profiles of phantom data simulating halo artifacts of the urine shift pattern: Random-corrected emission sinogram (*thick black solid line*), scatter sinogram (*red solid line:* TFS 4-mm, *green solid line:* TFS 2-mm, *blue solid line:* MCS 4-mm), and mask for TF-SSS (*red region:* for TFS 4-mm, *green region:* for TFS 2-mm). CT–/PET+ (A), CT+/PET– (B), CT+/PET+ (C).

**Additional file 2: Supplemental Fig.S2.** TFS 4-mm PET images when the urinary catheter is attached to the patient's skin surface (A,B). There were no halo artifacts.

## Abbreviations

MCS 4-mm: Monte Carlo-based single-scatter simulation using 4-mm voxels μ-maps
MC-SSS: Monte Carlo-based single-scatter simulation
NEMA: National Electrical Manufacturers Association
NSC: Non-scatter-correction
PSMA: Prostate-specific membrane antigen
SSS: Single-scatter simulation
TFS 2-mm: Tail-fitted single-scatter simulation using 2-mm voxel μ-maps
TFS 4-mm: Tail-fitted single-scatter simulation using 4-mm voxel μ-maps
TF-SSS: Tail-fitted single-scatter simulation
