# Supplementary figures and images for: Halo artifacts of indwelling urinary catheter by inaccurate scatter correction in 18F-FDG PET/CT imaging: incidence, mechanism, and solutions
Source: EJNMMI Phys. 2020 Nov 13;7:66. doi: 10.1186/s40658-020-00333-8 (PMC7666262; doi:10.1186/s40658-020-00333-8)

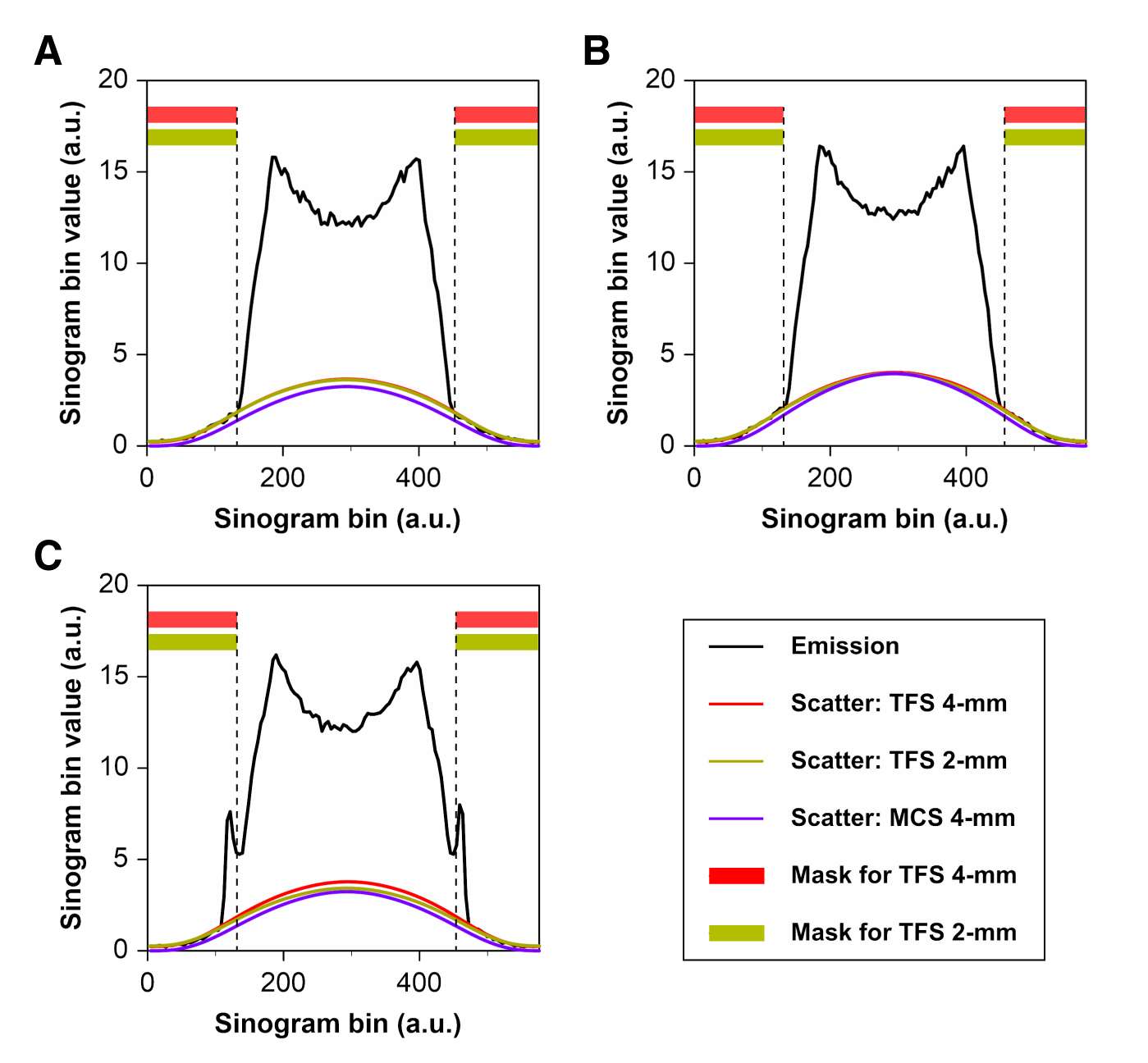

Supplement: Supplementary file 1 — Additional file 1: Supplemental Fig. S1. Sinogram profiles of phantom data simulating halo artifacts of the urine shift pattern: Random-corrected emission sinogram (thick black solid line), scatter sinogram (red solid line: TFS 4-mm, green solid line: TFS 2-mm, blue solid line: MCS 4-mm), and mask for TF-SSS (red region: for TFS 4-mm, green region: for TFS 2-mm). CT–/PET+ (A), CT+/PET– (B), CT+/PET+ (C). [file 40658_2020_333_MOESM1_ESM.tif]

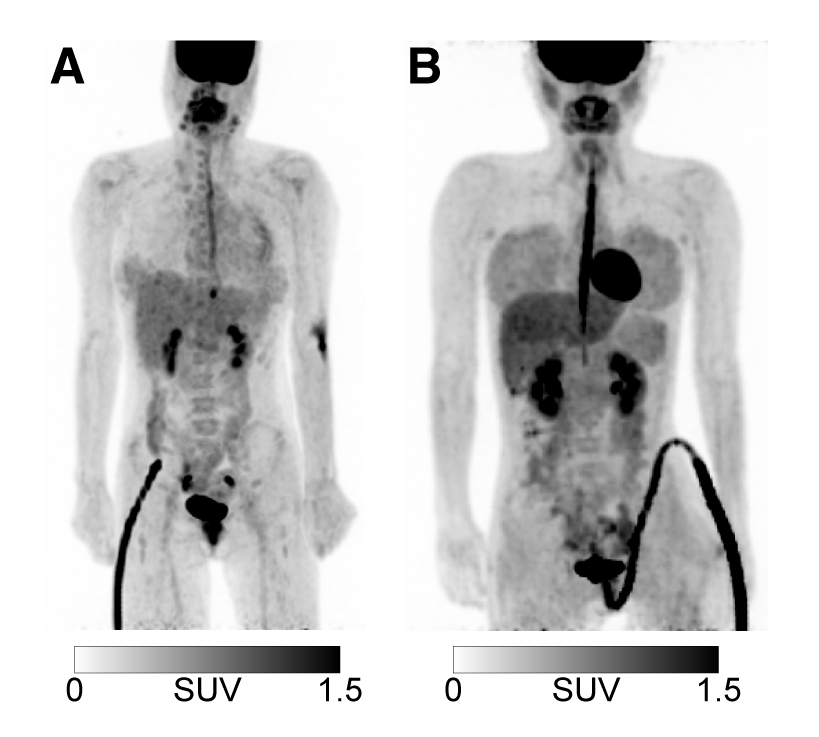

Supplement: Supplementary file 2 — Additional file 2: Supplemental Fig.S2. TFS 4-mm PET images when the urinary catheter is attached to the patient's skin surface (A,B). There were no halo artifacts. [file 40658_2020_333_MOESM2_ESM.tif]
